# TIMP-1-Mediated Chemoresistance via Induction of IL-6 in NSCLC

**DOI:** 10.3390/cancers11081184

**Published:** 2019-08-15

**Authors:** Wei Xiao, Lan Wang, John Howard, Ravindra Kolhe, Amyn M. Rojiani, Mumtaz V. Rojiani

**Affiliations:** 1 Department of Pathology, Medical College of Georgia-Augusta University, Augusta, GA 30912, USA; 2 Georgia Cancer Center, Augusta University, Augusta, GA 30912, USA; 3 Departments of Medicine, Medical College of Georgia, Augusta University, Augusta, GA 30912, USA

**Keywords:** TIMP-1, IL-6, chemoresistance, NSCLC, tumor microenvironment

## Abstract

Elevated tissue inhibitor of metalloproteinase-1 (TIMP-1) is a negative prognosticator in non-small cell lung carcinoma NSCLC patients. This study sought to identify mechanisms whereby TIMP-1 impacts anticancer therapy. Using NSCLC cells and their TIMP-1 knockdown clones, we examined the chemoresistance against two chemotherapeutic agents, Gemcitabine and Cisplatin, as identified by increased apoptosis in the knockdown clones. A bead-based cytokine screening assay identified interleukin-6 (IL-6) as a key factor in chemoresistance. Exogenous human recombinant rhTIMP-1 or rhIL-6 resulted in reduced apoptosis. IL-6 expression was closely correlated with TIMP-1 kinetics and was upregulated by the addition of exogenous TIMP-1 while TIMP-1 neutralizing antibodies delayed IL-6 elevation. IL-6 production was regulated by TIMP-1, exerting its effect via activation of downstream signal transducer and activator of transcription 3 (STAT3) signaling. Both molecules and their documented transcription factors were upregulated and activated in chemoresistant NSCLC cells, confirming the roles of TIMP-1 and IL-6 in chemoresistance. To examine the role of these genes in patients, survival data from lung adenocarcinoma (LUAD) patients was curated from the cancer genome atlas (TCGA) database. Kaplan-Meier analysis found that individuals expressing low TIMP-1 and IL-6 have a higher survival rate and that the two-gene signature was more significant than the single-gene status. We define for the first time, a regulatory relationship between TIMP-1 and IL-6 in NSCLCs, suggesting that the TIMP-1/IL6 axis may be a valuable prognostic biomarker. Therapeutic interventions directed at this dual target may improve overall prognosis while negatively affecting the development of chemoresistance in NSCLC.

## 1. Introduction

Lung cancer is responsible for almost one-quarter of all cancer deaths and remains the leading cause of both cancer incidence and mortality globally [1,2]. As the predominant component, non-small cell lung carcinoma (NSCLC) accounts for 87% of all lung cancers [1]. In clinical settings, systemic chemotherapy is the mainstay of treatment, particularly as the primary modality for patients with advanced disease. Even with radiation and surgery, chemotherapy remains a valuable adjuvant therapeutic consideration [3]. Although these patients clearly benefit from such chemotherapy, it rarely results in a cure with the majority of patients developing recurrences that are not only chemoresistant but are also often more aggressive [4]. 

Tissue inhibitors of metalloproteinase (TIMPs) are classically identified as tumor-inhibitory by virtue of their ability to curb matrix metalloproteinase (MMP)-dependent activities [5]. However, numerous studies have documented that high blood and tissue levels of TIMP-1 in cancer patients are associated with poor prognosis and decreased survival in many cancers, including NSCLC [6]. The MMP-independent activity of TIMP-1 provides the mechanisms for the tumor-promoting functions of TIMP-1 in angiogenesis, invasion, and metastasis [7].

Several recent studies have documented a potential chemoresistant function of TIMP-1 [8,9]. Our own studies have confirmed that the high expression of TIMP-1 results in activation of pro-survival and anti-apoptotic signaling through the extracellular-signal-regulated kinase (ERK) and BCL2 associated agonist of cell death (BAD) pathway, as well as the down-regulation of miR-125a-5p in NSCLCs [9,10]. Although the understanding of this function of TIMP-1 is starting to gain grounds, the precise mechanisms by which TIMP-1 causes chemoresistance remains elusive.

The tumor microenvironment (TME), harbors growth factors and cytokines that contribute to resistance to chemotherapy [11,12]. IL-6, a pluripotent cytokine in the TME, serves as such a potential contributor, through the activation of downstream signaling pathways [13]. Intriguingly, under specific conditions, IL-6 function in tumors appears to have a direct correlation with TIMP-1. It has been established that IL-6 controls TIMP-1 expression as an upstream regulator in various human stromal cells and malignant non-Hodgkin’s lymphoma [14,15]. A recent study has reported that IL-6 alone or in combination with TIMP-1, facilitated murine lymphoma resistance to chemotherapy-induced DNA damage via promotion of cell growth [16]. However, the bona fide relationship between TIMP-1 and IL-6 remains unclear.

In this study, we sought to determine the role of TIMP-1 in creating a chemoresistant niche and mediating chemoresistance in NSCLCs through modulating soluble factors in the surrounding environment. We report a critical role for TIMP-1 in this chemoresistance, acting by upregulating the production of cytokine IL-6 and activating activator protein 1 (AP-1) transcriptional activities. Our study has identified TIMP-1 as a potential determinant that modulates the tumor microenvironment, contributing to resistance against chemotherapy.

## 2. Materials and Methods

### 2.1. Cell Culture

Human NSCLC cell lines (A549 and H460) were purchased from American Type Culture Collection (ATCC, Manassas, VA, USA). A549- and H460-derived cell clones, encoding non-target scrambled shRNA (-NT) or TIMP-1-specific knockdown shRNA (-KD) sequences, characterized in our previous studies [10] were used. A549 and its clones were cultured in F-12K medium, and H460 and its clones were grown in RPMI Medium 1640 (Sigma-Aldrich, St. Louis, MO, USA) as per ATCC recommendations. Cells were cultured under normoxic (20% O_2_ with 5% CO_2_), or under hypoxic conditions as indicated (1% O_2_ with 5% CO_2_). To determine the relationship between TIMP-1 and IL-6, we re-constructed TIMP-1 KD, IL-6 KD, and NT clones with shRNA lentiviral particles (Santa Cruz Biotechnology, Dallas, TX, USA), respectively, as per the manufacturer’s instructions. All human cell lines were authenticated using short tandem repeat (STR) (or single nucleotide polymorphism (SNP) profiling within the last three years and are mycoplasma-free cells. 

### 2.2. Preparation of Conditioned Media (CM)

Cells were normally grown in complete media for 48 h. Culture media supernatants were collected, and centrifuged at 2000 rpm for 10 min, at 4 °C. Supernatants were stored at −80 °C until utilized.

### 2.3. Real-Time Quantitative RT-PCR (qRT-PCR)

Total RNA was prepared using phenol-chloroform extraction, followed by RNA purification with PureLink^®^ RNA Mini Kit (Thermo Fisher Scientific, Waltham, MA, USA) and DNase treatment with PureLink^®^ DNase (Invitrogen, Carlsbad, CA, USA). Complementary DNA (cDNA) was synthesized from 1 µg mRNA using iScript cDNA Synthesis Kit (Bio-Rad, Hercules, CA, USA). For qRT-PCR), gene mRNA was measured in CFX Connect Real-Time PCR System with iQ SYBR Green Supermix (Bio-Rad) and specific primers (Integrated DNA Technologies, Coralville, IA, USA). β-Actin was used as a reference gene, and each measurement was done twice in triplicates. All primer pairs are shown in Table S1.

### 2.4. Immunoblotting

For cell extracts, cells were harvested at specific time-points and lysed in ice-cold RIPA buffer (TEKnova, Hollister, CA, USA) supplemented with protease and phosphatase inhibitors and then briefly sonicated. For secreted TIMP-1, the same number of cells were seeded into equal volumes of culture medium. Supernatant conditioned media were collected at specific time points. SDS-PAGE and western blotting were as described previously [7,9]. In some experiments, protein levels were quantified by measuring protein band intensities with the ImageJ program, normalized to the loading control.

All antibodies are listed in Table S2. All original immunoblots are available for review in Figure S6.

### 2.5. Apoptosis Assay

Cell apoptosis and death was evaluated using Allophycocyanin (APC)-conjugated annexin V/propidium iodide (PI) (BD PharMingen) staining followed by flow cytometry as previously described [17]. Chemotherapeutic agents, Cisplatin (Tocris Bioscience, Bristol, UK) or Gemcitabine (TCI America, Portland, OR, USA), were added into cultures and incubated for 48 to 72 h as indicated. In the apoptosis rescue assay, rhTIMP-1 or rhIL-6 (R&D Systems) were added at the start. Cells were harvested and washed with Annexin V binding buffer (BD Pharmingen, CA, USA), and then incubated with APC-conjugated Annexin V for 30 min. Propidium iodide (PI) was added 5 min prior to flow cytometry. Data were analyzed using the FlowJo.V10 software (FlowJo, Ashland, OR, USA). 

### 2.6. ELISA

Supernatants were collected at specific time points. In a blocking assay, neutralizing antibody against human-TIMP-1 or goat IgG (R&D Systems) was added into cell culture at the beginning. Quantitative detection of human IL-6 was performed with Human IL-6 ELISA MAX™ Deluxe kit (BioLegend, San Diego, CA, USA) as instructed.

### 2.7. Bead-Based Cytokine Immunoassay

The measurement of soluble factor levels in culture supernatants was performed using a 12-plex human Custom Panel (LEGENDplex, Biolegend, San Diego, CA, USA) as per the manufacturer’s instructions. The capture beads measured human-derived TGFβ, IL-1β, IL-1α, GM-CSF, G-CSF, IL-6, HGF, IFNα2, IL-12p70, VEGFα, IL-10, and TIMP-1. Data were collected on a FACS Calibur two-laser flow cytometer (Beckton Dickinson, Franklin Lakes, NJ, USA) and analyzed using the LEGENDplex Data Analysis Software (Biolegend).

### 2.8. TCGA Analysis

TCGAbiolinks was used to download clinical and gene expression data from harmonized GDC datasets (http://portal.gdc.cancer.gov) [18]. The package was installed on R 3.5.2 (R Core Team, Vienna, Austria). FPKM RNA-seq harmonized data (aligned to the GRCh38 reference genome) were filtered to retain “non-formalin-fixed paraffin embedded (FFPE)” samples with a single representative aliquot per participant. In case of duplicate aliquots, a choice was made to select the aliquot with the higher plate number (based on the barcode). The FPKM values were converted to transcripts per million (TPM) as they are more suitable to compare across the samples than the FPKMs [19]. To normalize the expression data for each gene, we calculated z-scores across all patients in the dataset. To generate multiple gene signatures, the mean z-scores for involved genes was used. For KM analysis, patients were divided on the basis of quartiles. Survival curves were plotted from the Kaplan-Meier estimates via the TCGAbiolinks R package. 

### 2.9. Statistical Analysis

Experimental data were expressed as the mean ± standard deviation (SD) using the GraphPad Prism 7 software (GraphPad Software, San Diego, CA, USA). The analysis of significance was performed using two-tailed *Student’s t*-test. Survival curves were estimated by the Kaplan-Meier method with the log-rank test using R. In all the figures, statistical significance is denoted as follows: * *p* < 0.05; ** *p* < 0.01; *** *p* < 0.001

## 3. Results

### 3.1. IL-6 Is a Determinant in TIMP-1-Mediated Chemoresistance

**Figure 1:**
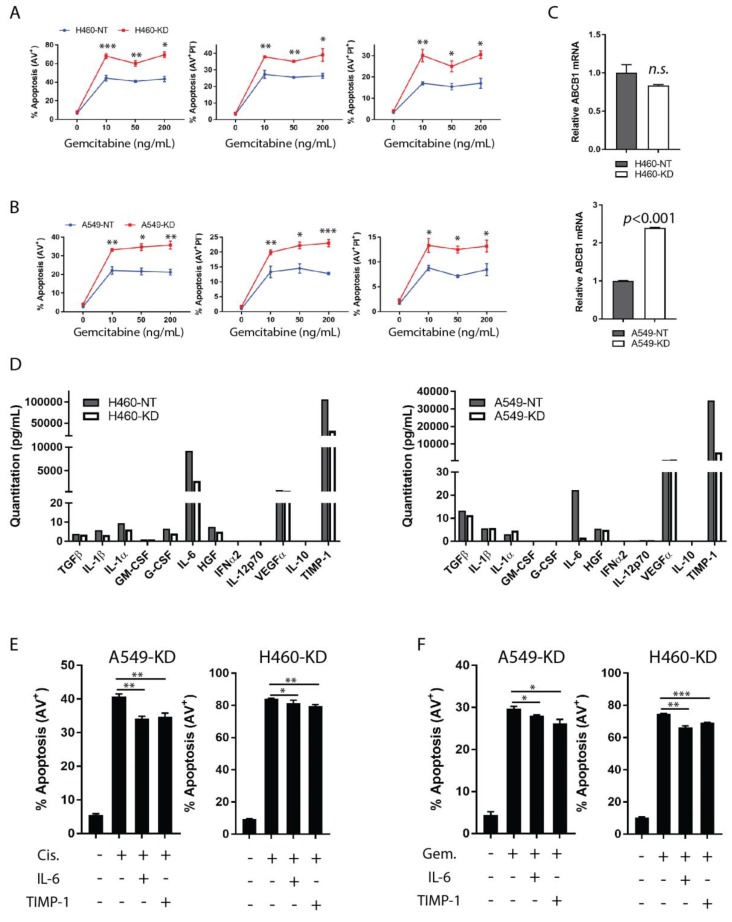
IL-6 is a determinant in TIMP-1-mediated chemoresistance. (**A**,**B**) Human NSCLC cells (A549 and H460) encoding non-target scrambled shRNA (NT) sequence, or TIMP-1-specific knockdown shRNA (KD) sequence were seeded in 24-well plates (3 × 10^4^/well). At log-phase, cells were treated with variable doses of Gemcitabine as indicated. All floating and adherent cells were collected at 72 h post-treatment, stained with Annexin V and PI and analyzed by flow cytometry. Statistical analysis of total apoptosis (Annexin V^+^), early apoptosis (Annexin V^+^ PI^−^), and apoptotic cell death (Annexin V^+^ PI^+^) is shown for H460-derived cells (**A**) and A549-derived cells (**B**). (**C**) Total RNA was extracted from NT and KD clones of A549 and H460. Real-time quantitative RT-PCR was performed to measure the mRNA transcripts of the ABCB1 gene. (**D**) H460- (left panel) or A549- (right panel) NT and KD clones suspended in complete fresh medium were cultured for three days. Supernatants collected at 72 h were titrated for absolute concentrations of the following: TGF-ß, IL-1ß, IL-1a, GM-CSF, G-CSF, IL-6, HGF, IFNα2, IL-12p70, VEGF-A, IL-10, and TIMP-1, with a multiplex human custom panel by Bead-based cytokine immunoassay (BCA). (**E**,**F**) A549- or H460-derived TIMP-1 KD clones were cultured in complete medium without, or supplemented with exogenous rhIL-6 (25 ng/mL) or rhTIMP-1 (25 ng/mL) in the presence or absence of chemotherapeutic agents (25 µg/mL Cisplatin or 10 ng/mL Gemcitabine), respectively, for 72 h. Apoptosis was analyzed by Annexin V and PI staining, followed by flow cytometry. Statistical significance is denoted as follows: * *p* < 0.05; ** *p* < 0.01; *** *p* < 0.001.

To address the effect of TIMP-1 in chemotherapy, NSCLC A549 and H460 and their TIMP-1 non-target (NT) and knock-down (KD) clones were employed (Supplementary Figure S1A). The knockdown of the TIMP-1 gene does not affect the proliferation rate in both NSCLC cell lines [10]. These cells were analyzed for apoptosis after treatment with Gemcitabine or Cisplatin, which are routinely used as frontline chemotherapeutic agents for lung carcinomas. The TIMP-1 KD clones of both H460 and A549 were more sensitive to apoptosis induced by Gemcitabine compared to the NT controls as determined by flow cytometric analysis (Figure 1A,B) and (Supplementary Figure S1B,C). As seen in Figure 1A,B, both H460-KD and A549-KD cells exhibited higher percentages of early apoptosis (Annexin V^+^ PI^−^) and late apoptotic cell death (Annexin V^+^ PI^+^) than non-target controls. Similar results were observed when cells were treated with Cisplatin, although A549-KD showed higher sensitivity to Cisplatin only during early apoptosis, and H460-KD showed higher sensitivity to Cisplatin at later stages of apoptotic cell death (Supplementary Figure S2A,D). These observations indicate that the knockdown of TIMP-1 increased the sensitivity of these cells to chemotherapy, making them vulnerable to chemo-induced apoptosis, supporting the contention that TIMP-1 expression contributes to chemoresistance/tolerance. 

Upregulation of the multidrug resistance (MDR) gene is most frequently associated with chemoresistance where overexpression of ATP-binding cassette (ABC) transporters facilitate the efflux of drugs across cell membranes [20]. Hence, as a first step to dissecting the chemoresistant mechanism of TIMP-1, we investigated the relative mRNA level of ABCB1 (MDR1). As shown in Figure 1C, the ABCB1 gene transcription remained unaltered in the H460 cell line under study upon TIMP-1 KD (upper panel), but conversely, was increased in TIMP-1 KD cells of A549 (lower panel). This data suggests that TIMP-1-mediated chemoresistance must be attributed to factors other than MDR1 overexpression. 

Recognizing that the MMP-independent activity of TIMP-1 is a signaling function, we sought to determine any contribution of altered cytokine activity towards the anti-apoptotic function of TIMP-1. We executed a screening of soluble factors in culture medium supernatants by a bead-based cytokine immunoassay. IL-6 production decreased dramatically (more than two fold), in culture medium supernatants from TIMP-1 KD clones of H460 cells (upper panel) and A549 cells (lower panel), compared to corresponding non-targeted controls (Figure 1D). In contrast, other detected factors, including inflammatory cytokines, colony-stimulating factors (CSF), and growth factors (GF), did not show any significant alterations between KD and NT clones. Further verification of mRNA levels of selected cytokines demonstrated that TIMP-1 knockdown specifically down-regulated the IL-6 transcript, but not that of IFNγ, TGFβ, or IL-10 (Supplementary Figure S3A,B, (data not shown) for undetectable IL-10). Collectively, the results of this screening and mRNA verification confirms IL-6 to be the major cytokine altered upon TIMP-1 modulation. 

IL-6 is well known as a pluripotent cytokine involved in multiple biological functions in the TME, including drug resistance [21]. To investigate the hypothesis that IL-6 contributes to TIMP-1-mediated chemoresistance in NSCLCs, we carried out an apoptosis rescue assay to determine if exogenous rhIL-6 or rhTIMP-1 could protect NSCLC cells from chemotherapy-induced apoptosis. Our data indicate that exogenous rhIL-6 (25 ng/mL) did not affect the proliferation rate of knockdown clones for both NSCLC cell lines (Supplementary Figure S5A). Utilizing TIMP-1 KD clones of H460 or A549 cells (with low TIMP-1/IL-6 expression), we determined the response to chemotherapy in the presence of exogenous rhIL-6 or rhTIMP-1. The results show that TIMP-1 KD clones of both cell lines showed reduced Cisplatin- or Gemcitabine-induced apoptosis (Annexin V^+^) when they were simultaneously exposed to either exogenous rhIL-6 or rhTIMP-1 (Figure 1E,F).

### 3.2. IL-6 Expression Correlates Closely with TIMP-1 Kinetics

**Figure 2:**
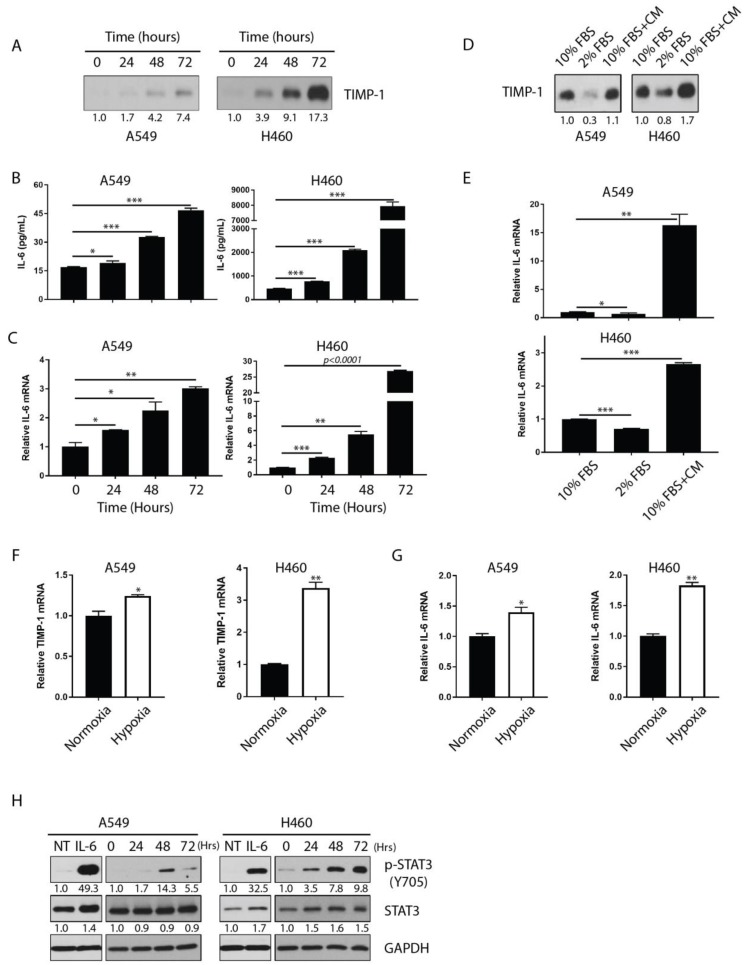
IL-6 expression is closely correlated with TIMP-1 production. (**A**) NSCLC cells (A549 and H460) were cultured in complete medium for three consecutive days. Supernatants were collected at 0, 24, 48, and 72 h. Cell attachment time was defined as the 0 h time point. Equal volumes of supernatants were loaded for human TIMP-1-specific immunoblotting analysis under reduced conditions. (**B**) Supernatants were used to measure IL-6 production at the same time-points by ELISA. (**C**) IL-6 mRNA transcripts were also measured in cells at these time-points by real-time RT-PCR. (**D**) A549 and H460 cells were cultured in normal complete medium (10% FBS), FBS-reduced medium (2% FBS), and conditioned culture medium (1:1 mixture of 10% FBS medium and conditioned medium, 10% FBS + CM) for three days. Equal volumes of supernatants were loaded for human TIMP-1-specific immunoblotting analysis. (**E**) IL-6 mRNA transcripts were measured in cells collected from different conditions as above in D by RT-PCR. TIMP-1- (**F**) and IL-6 (**G**) mRNA transcripts were measured in normoxia (20% O_2_) or hypoxia (1% O_2_) cell cultures at 72 h by RT-PCR. (**H**) NSCLC cells grown in regular cultures for three days, following which whole cell lysates extracted at 0, 24, 48, and 72 h and analyzed for activation of STAT3 signaling by immunoblotting. Cells treated with or without exogenous IL-6 (100 ng/mL) served as positive controls for STAT3 signaling activation. Statistical significance is denoted as follows: * *p* < 0.05; ** *p* < 0.01; *** *p* < 0.001.

In order to establish a bona fide relationship between TIMP-1 and IL-6, we determined the expression profiles of TIMP-1 and IL-6 in NSCLC cell lines under different experimental conditions, including a kinetic study under normal culture conditions. TIMP-1 protein accumulated in supernatants over the incubation period in culture (Figure 2A). There was a simultaneous increase in the levels of IL-6 mRNA and protein consistently over the time course of culture in both cell lines, which correlated positively with the dynamic changes in TIMP-1 (Figure 2B,C). 

To further corroborate this positive correlation between IL-6 and TIMP-1, we determined the levels of TIMP-1 and IL-6 under inductive conditions, such as reduced-serum (2% FBS) condition or with conditioned-media (CM) from previous cultures. Compared to normal culture conditions (10% FBS), NSCLC cells in 2% reduced-serum-conditions secreted lower levels of TIMP-1 protein into the media, whereas higher levels of TIMP-1 protein were produced when the cells were treated with CM from prior cultures (Figure 2D). The IL-6 mRNA transcript showed a positive correlation with the dynamics of TIMP-1 production and release in the media, thus confirming their strong and distinct positive correlation (Figure 2E).

Solid tumors often develop a hypoxic milieu within certain regions of the tumor. TIMP-1 regulation has previously been associated with hypoxia-responsive element reporter activity [22]. To examine alterations in TIMP-1 expression, we challenged NSCLC cells with a hypoxic environment (1% O_2_). Both cell lines showed an increase in TIMP-1 mRNA levels under hypoxic compared to normoxic conditions (Figure 2F). Again, IL-6 mRNA levels also showed a corresponding similar increase to TIMP-1 mRNA level under hypoxia (Figure 2G). These modulations point to the fact that there is, a positive relationship between TIMP-1 and IL-6.

Although our results so far demonstrate that IL-6 expression is linked to TIMP-1 modulation, we also needed to determine whether the expressed IL-6 is functional in NSCLCs. To address this, we investigated the phosphorylation of STAT3, a key downstream molecule in IL-6 signaling. Figure 2H shows that STAT3 continued to be phosphorylated over the time course of IL-6 accumulation (as seen above in Figure 2B,C) and; therefore, was activated by IL-6 in both NSCLC cell lines (Figure 2H). This confirms that TIMP-1-regulated IL-6 is indeed functional in NSCLCs through the activation of its downstream signaling pathway.

### 3.3. TIMP-1 Regulates IL-6 Expression in NSCLCs

**Figure 3:**
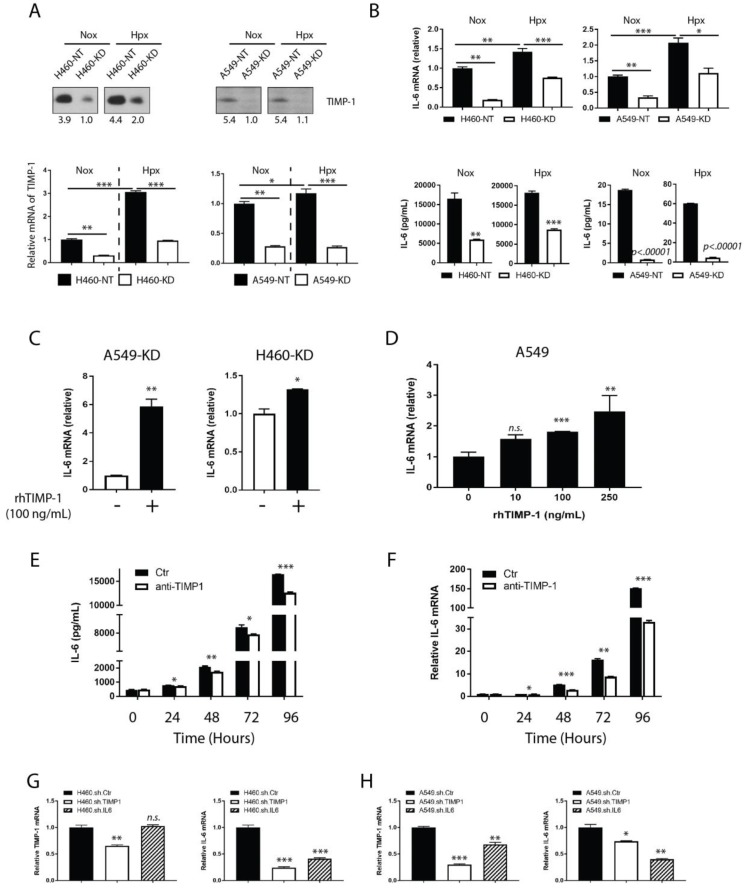
TIMP-1 regulates IL-6 expression. A. H460- or A549-derived TIMP-1 NT and KD clones were cultured under normoxic (20% O_2_) or hypoxic (1% O_2_) conditions, respectively, at 72 h. (**A**) The presence of TIMP-1 protein (upper panel) in culture supernatants was detected by immunoblot analysis. TIMP-1 mRNA transcripts (lower panel) were measured by RT-PCR. (**B**) IL-6 mRNA transcripts (upper panel) were measured in H460- or A549-derived clones under the same culture conditions as above, by RT-PCR. IL-6 protein levels (lower panel) in supernatants from the above cell cultures were titrated by ELISA. (**C**) A549-KD, or H460-TIMP-1 KD clones were treated with exogenous rhTIMP-1 (100 ng/mL) for 24 h. IL-6 mRNA transcripts were measured by RT-PCR. (**D**) A549 cells were treated with exogenous rhTIMP-1 at different doses (ranged from 0 ng/mL to 250 ng/mL) for 24 h. Cell lysates were used to measure IL-6 mRNA transcripts by RT-PCR. (**E**,**F**) H460 cells were cultured in complete media for four days. In parallel, one group of cells was treated with neutralizing anti-TIMP-1 antibodies (500 ng/mL), while another group was treated with goat IgG as a control. Culture medium supernatants were collected in pairs at 0, 24, 48, 72, and 96 h, respectively. The IL-6 protein was also titrated by ELISA. The IL-6 mRNA level was determined in cell pellets in the above conditions by RT-PCR. Cell attachment time was defined as the 0 h time-point. (**G**,**H**) To determine the bona fide relationship between TIMP-1 and IL-6, TIMP-1 KD, IL-6 KD, and NT clones were constructed with concentrated, transduction-ready lentiviral particles containing target-specific shRNA-encoding constructs in H460 and A549 cells, respectively. TIMP-1 and IL-6 mRNA levels were determined by RT-PCR. Statistical significance is denoted as follows: * *p* < 0.05; ** *p* < 0.01; *** *p* < 0.001.

The literature documents that TIMP-1 is the downstream molecule of IL-6 [14,15]. However, our data raise the question that this may indeed be reversed, i.e., IL-6 is downstream of TIMP-1 in NSCLC and that TIMP-1 regulates IL-6 in NSCLCs. To validate this hypothesis, we interrogated H460- or A549-derived clones to determine TIMP-1 transcripts under either normoxic (20% O_2_) or hypoxic (1% O_2_) conditions. TIMP-1 mRNA and protein levels decreased significantly in TIMP-1 KD clones compared to NT clones under both conditions (Figure 3A). Hypoxia induced TIMP-1 upregulation in both NT and KD clones compared to normoxia, with a pronounced effect in H460-derived clones. TIMP-1 levels have been shown to increase under hypoxia [23]. Correspondingly, IL-6 mRNA and protein levels showed a positive correlation with TIMP-1 expression. Again, this held true for both NT and KD clones with an increase under hypoxic conditions for IL-6 expression in both mRNA and protein levels compared to normoxia (Figure 3B).

We then sought to determine if exogenous TIMP-1 affected IL-6 expression. To negate any influence of endogenous TIMP-1, KD clones of TIMP-1 (with low basal TIMP-1 level) were treated with rhTIMP-1. This treatment showed an elevated response of IL-6 mRNA in both clones, after exogenous TIMP-1 treatment (Figure 3C). Additionally, we treated parental A549 cells (with a relatively low TIMP-1 expression, Supplementary Figure S4A,B), with different doses of exogenous rhTIMP-1. The result indicated that the transcription of the IL-6 gene was regulated by exogenous rhTIMP-1 in a dose-dependent manner (Figure 3D). For H460 cells (with a relatively high TIMP-1 expression -Supplementary Figure S4A,B), we applied neutralizing anti-TIMP-1 antibodies to specifically block secreted TIMP-1 during culture, and compared the IL-6 mRNA and protein levels with the immunoglobulin G (IgG) control group. Over a 96-hour period, neutralizing endogenous TIMP-1 remarkably decreased the IL-6 protein and mRNA levels compared to coinstantaneous control, although a high dose of neutralizing anti-TIMP-1 antibodies did not completely inhibit the elevation of IL-6 (Figure 3E,F). Overall, the blockade of extracellular TIMP-1 delayed the upregulation of IL-6 expression, which provided further corroboration of the TIMP-1 controlling IL-6.

Our results demonstrate that IL-6 expression was modulated by endogenous, as well as exogenous TIMP-1, but it was not known if IL-6 affects TIMP-1 expression in NSCLCs. To address this question, we carried out a parallel reconstruction of TIMP-1 or IL-6 knockdown clones with concentrated, transduction-ready lentiviral particles containing target-specific shRNA-encoding constructs in H460 and A549 cells. The results reconfirmed that genetically silencing the TIMP-1 gene significantly down-regulated IL-6 mRNA transcription in both NSCLC cell lines, whereas the knockdown of IL-6 did not change TIMP-1 transcription in H460, and caused only limited downregulation in TIMP-1 transcripts in A549 cells (Figure 3G,H). Therefore, TIMP-1 regulation of IL-6 expression in NSCLCs is evident. However, the effect of IL-6 on TIMP-1 expression remains inconsistent between the two cell lines. The change seen in the A549 cells even though relatively small is statistically significant and; therefore, may still be minimally relevant. 

### 3.4. Involvement of Transcription Factor AP-1 in the TIMP-1 Regulation of IL-6

**Figure 4:**
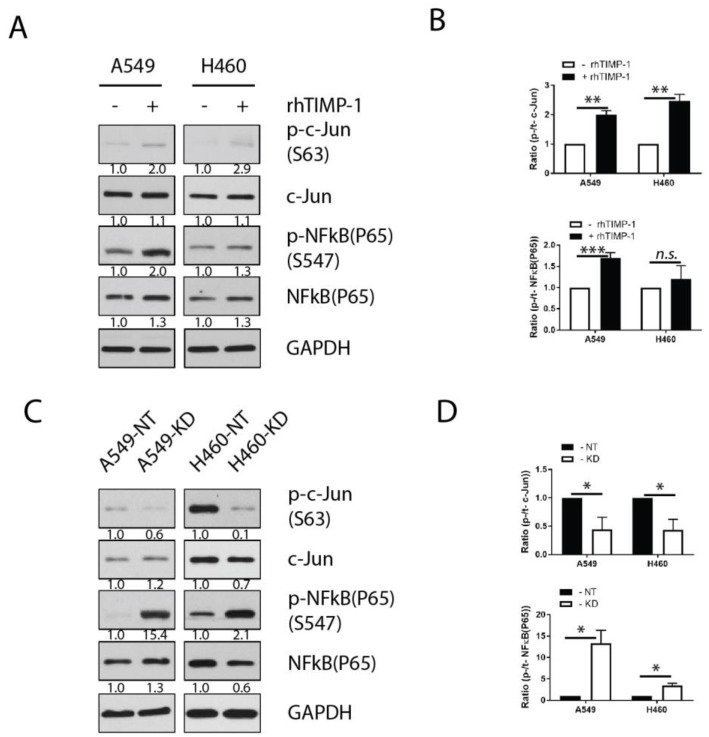
AP-1 transcriptional factor participates in TIMP-1-regulated IL-6 expression. (**A**) A549 and H460 cells were cultured in complete medium and treated with exogenous rhTIMP-1 for one hour. Whole-cell lysates were analyzed for transcription signaling, as indicated by immunoblotting. (**B**) The band intensity of relevant protein (NFκB (P65) and AP-1 (c-Jun)) was normalized to that of the reference gene by the ImageJ program. Relative ratios of phosphorylated signal to total signal are presented. (**C**) A549- or H460-derived NT and KD clones were seeded into flasks at 70–80% confluency and grown in complete medium with 10% FBS. Cells were rinsed once and replaced with warm FBS-free medium for another six hours prior to lysis. Whole-cell lysates were analyzed for transcription signaling as indicated by immunoblotting. (**D**) The band intensity of relevant proteins was normalized to that of the reference gene by the ImageJ program. Relative ratios of phosphorylated signal to total signal are shown. Statistical significance is denoted as follows: * *p* < 0.05; ** *p* < 0.01; *** *p* < 0.001.

NFκB (nuclear factor *kappa*-light-chain-enhancer of activated B cells) and activator protein-1 (AP-1) are important inducible transcription factors involved in IL-6 expression and regulation [24]. However, transcription factor(s) involved in TIMP-1 regulation of IL-6 in NSCLCs remains unknown. To address this, parental cells (A549 and H460) were treated with exogenous rhTIMP-1 for an hour, followed by the detection of the phosphorylated and total expression of subunits of transcription factors NFκB and AP-1. We normalized all signals to the corresponding reference gene by densitometry. The relative ratios of phosphorylated signal to total signal indicated that AP-1 (c-Jun) was activated in both NSCLC cell lines following the addition of exogenous TIMP-1 to the cell culture (Figure 4A,B). The subunit P65 of NFκB was activated in A549 cells, but not in H460 cells, when cells were treated with rhTIMP-1 (Figure 4A,B). 

In a similar approach, we assessed these two transcription factors in stable TIMP-1 KD and corresponding NT controls. For the AP-1 transcription factor, we found the relative ratios of the phosphorylated c-Jun to total c-Jun to be significantly decreased in TIMP-1 KD clones in comparison with NT clones (Figure 4C,D). However, for NFκB transcription factor, relative ratios of phosphorylated P65 to total P65 increased significantly in KD clones (Figure 4C,D). The activated status of AP-1 (c-Jun) is correlated with TIMP-1-mediated IL-6 alteration; thereby confirming that AP-1 is the most relevant potential transcription factor involved in the TIMP-1 regulation of IL-6. 

### 3.5. Chemoresistant NSCLC Cells Upregulate TIMP-1 and IL-6 and THEIR Transcriptional Activity

**Figure 5:**
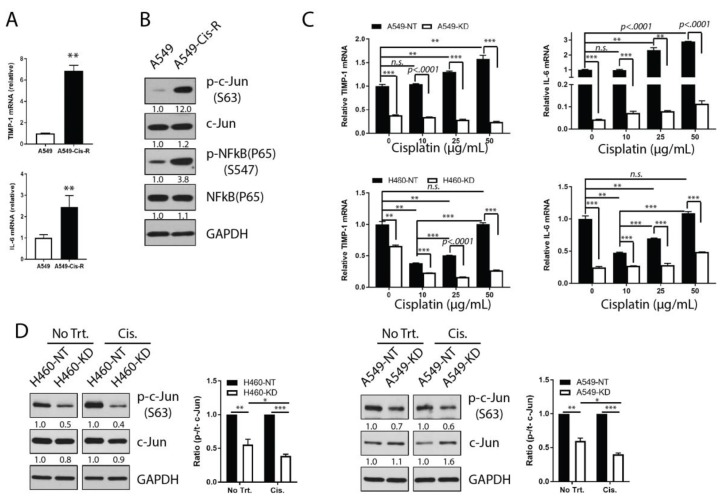
NSCLC cells increased their tolerance to two frontline chemotherapy drugs via upregulation of TIMP-1 and IL-6. Cisplatin-resistant A549 cells (A549-Cis-R) were established by treatment with gradient doses of Cisplatin and finally maintained in complete medium supplemented with 15 µg/mL of Cisplatin. (**A**) Whole cell lysates from parental A549- and A549-Cis-R as well as TIMP-1 and IL-6 mRNA were analyzed by RT-PCR. (**B**) Whole-cell lysates were analyzed for transcription signaling, as indicated by immunoblotting. GAPDH was the reference loading control. (**C**) A549- or H460-derived shRNA-encoding cells (A549-NT, A549-KD, H460-NT, and H460-KD) were treated with Cisplatin at different doses as indicated for 72 h. TIMP-1 and IL-6 mRNA expression was measured by qRT-PCR. (**D**) The above cells were treated with Cisplatin for one hour. The response of AP-1 transcriptional signal was detected by immunoblotting. The band intensity of the relevant protein was normalized to that of the reference gene by the ImageJ program. The relative ratios of phosphorylated signal to total signal are presented. Statistical significance is denoted as follows: * *p* < 0.05; ** *p* < 0.01; *** *p* < 0.001.

To unequivocally demonstrate the contributions of TIMP-1 and IL-6 to chemoresistance, we generated chemoresistant clones of NSCLC cells by a continuous escalation of drug dose over a span of three months (Supplementary Figure S5B). Compared to parental cells, Cisplatin-resistant A549 cells (A549-Cis-R) thus obtained, had a six-fold higher level of TIMP-1 mRNA and greater than a two-fold higher level of IL-6 mRNA (Figure 5A). This evidence of upregulated TIMP-1 and IL-6 in chemoresistant NSCLCs conformed closely to our above results of TIMP-1 and IL-6 rescuing chemo-induced apoptosis. We further determined transcriptional activities related to IL-6 regulation in chemoresistant cells and parental cells. Immunoblot results showed that the Cisplatin-resistant NSCLC cells showed increased activation of their signaling of c-Jun and NFκB compared to parental cells. This is observed as increased phosphorylation of these proteins, indicating that increased transcriptional activities might control IL-6 expression in Cisplatin-resistant NSCLC cells (Figure 5B).

So far, we do not know what the impact of TIMP-1 is on IL-6 regulation under chemotherapeutic treatment. To address this question, we determined the transcriptions of TIMP-1 mRNA and IL-6 mRNA in TIMP-1 KD clones and NT controls of NSCLC cells at 72 h post Cisplatin treatment. In NT clones, Cisplatin-induced TIMP-1 transcription in a dose-dependent manner in both NSCLC cells (Figure 5C). Although in H460 cells, TIMP-1 mRNA levels decreased in response to low dose (10 µg/mL) of Cisplatin, it was subsequently upregulated with an increasing dose of Cisplatin. In TIMP-1 KD clones, TIMP-1 mRNA consistently maintained a relatively low expression level compared to NT clones, and its transcription actually decreased in KD cells in response to different doses of Cisplatin (Figure 5C). Concomitantly, Cisplatin-induced IL-6 mRNA expression behaved in a similar manner as TIMP-1 mRNA transcription in NT clones of both NSCLC cells. However, in both TIMP-1 KD clones, Cisplatin still induced IL-6 mRNA transcription in a dose-dependent manner (Figure 5C). There was a positive correlation between TIMP-1 and IL-6 in NT clones in response to chemotherapy, but upregulation of IL-6 mRNA by chemotherapeutic treatment did not depend on the transcription of TIMP-1 mRNA since TIMP-1 mRNA levels decreased in KD clones responding to chemotherapy (Figure 5C).

To explore the regulation of transcription factors in TIMP-1 regulation of IL-6 against chemotherapy, we used TIMP-1 KD and NT clones to check AP-1 activity following treatment with chemotherapeutic drugs, since c-Jun, but not NFκB, had shown consistent activation with IL-6 regulation. When cells were treated with Cisplatin for one hour, phosphorylated c-Jun was activated by Cisplatin treatment in NT clones, but inactivated in KD clones, especially for H460 derived clones (Figure 5D). To compare the responsiveness of chemo-treatment between NT and KD clones, all signals were normalized to the reference loading control gene (GAPDH). Relative activated status of c-Jun transcription factor was measured by means of the ratio of the phosphorylated level to total protein level (p-/t-c-Jun). Overall, the degree of difference in ratio of p-/t-c-Jun between NT and KD clones increased substantially when the cells were treated with chemotherapeutic drugs based on signal quantification (Figure 5D). This suggests that knockdown of TIMP-1 reduced the responsiveness of c-Jun transcriptional activity in IL-6 regulation by chemotherapeutic treatment.

### 3.6. Co-Expression of TIMP-1 and IL-6 Inversely Correlates with the Survival of NSCLC Patients

**Figure 6:**
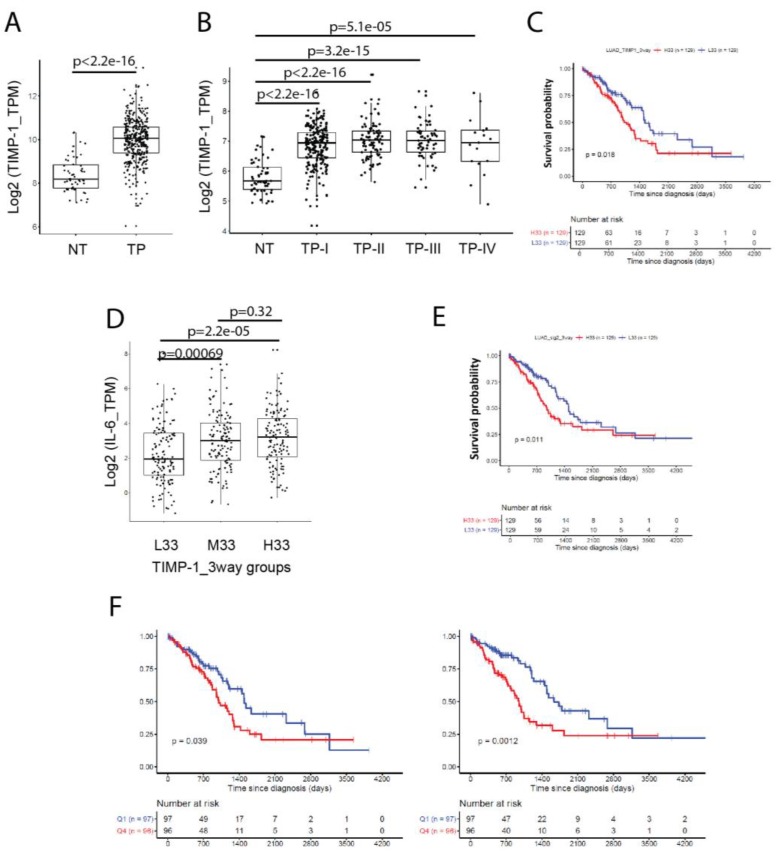
Meta-data analysis of TIMP-1 and IL-6 coexpression and the relationship with survival in clinical lung adenocarcinoma (LUAD) databases. (**A**) Mining the clinical lung adenocarcinoma of the Cancer Genome Atlas network (TCGA) data set (http://www.oncolnc.org) and comparison of TIMP-1 expression between tissues of primary tumor (TP) and adjacent normal tissues (NT). (**B**) Comparison of TIMP-1 expression in TP tissues and NT at four different stages. (**C**) Three-way grouping of LUAD patients based on TIMP-1 gene expression levels, and Kaplan–Meier survival analysis of patient sub-populations with high (H33) and low (L33) levels of TIMP-1 expression. (**D**) Comparison of IL-6 expression among patient sub-populations with different TIMP-1 expression levels. (**E**) Three-way grouping of LUAD patients based on TIMP-1 and IL-6 co-expression levels (two-gene signature) and Kaplan-Meier survival analysis of patient sub-populations with different levels (H33 and L33) of TIMP-1 and IL-6 co-expression. (**F**) Four-way grouping of LUAD patients based on TIMP-1 gene expression levels (single gene, left panel) or TIMP-1 and IL-6 co-expression levels (two-gene signature) (right panel), respectively, and Kaplan–Meier survival analysis of patient sub-populations with high (Q4) and low (Q1) levels of TIMP-1 expression or TIMP-1 and IL-6 co-expression. Statistical significances (log-rank test) were indicated (*p* < 0.05 is considered to be significant).

In patients, higher TIMP-1 mRNA expression level has been associated with worse prognosis in various cancers, including NSCLC [25]. Following data mining of gene expression in tissues of primary lung adenocarcinoma (TP, *n* = 405) and paired normal tissues (NT, *n* = 55) from 387 patients of “white” racial background from the TCGA database, we confirmed that the mRNA levels of TIMP-1 expression were significantly higher in TP tissues than NT (Figure 6A). We also analyzed the expression of TIMP-1 in different tumor stages of lung adenocarcinoma (LUAD). There was no significant difference for TIMP-1 expression between the four clinical stages of tumor progression. Moreover, the expression in all TP stages remained significantly higher as compared to NT (Figure 6B). To validate the effect of a single gene TIMP-1 expression on the survival of LUAD patients, 387 patients were grouped into tertiles based on TIMP-1 gene expression levels, and the Kaplan-Meier survival analysis was performed. L33 represents bottom the 33% patient population with the lowest TIMP-1 expression, while H33 represents top 33% patients with the highest TIMP-1 expression. By comparing these two groups, the Kaplan-Meier plot shows that patients with the lowest expression of TIMP-1 had a significantly (*p* = 0.018) higher survival probability, but patients with the highest TIMP-1 expression have a lower probability of surviving (Figure 6C).

Furthermore, we analyzed the IL-6 expression among three TIMP-1-expressing groups to see if there is any correlation between these two genes in LUAD. The level of IL-6 expression in L33 group with a relatively lower level of TIMP-1 was significantly lower than that in other two groups with relatively higher TIMP-1 expression, although there was no significant difference for IL-6 expression between the M33 and H33 groups (Figure 6D). This data still suggests that a positive correlation between IL-6 and TIMP-1 expression exists in NSCLC patients. 

To test the correlation between survival of LUAD patients and combined expressions of TIMP-1 and IL-6, we generated a two-gene signature. Expressions for each gene were z-transformed across all the patients, and then z-scores for TIMP-1 and IL-6 were averaged. We re-grouped the above-mentioned LUAD patients into another three sub-populations, and performed Kaplan–Meier analysis to determine if combining the lower co-expression of TIMP-1 and IL-6 improves the predictive value of patients’ survival. As described previously, L33 represents the bottom 33% patient population with the lowest score of the two-gene signature, while H33 represents the top 33% patients with the highest signature score (Figure 6E). The Kaplan–Meier plot indicates that the bottom L33-signature patients have a higher survival probability than patients with high H33-signature, improving the *p*-value (*p* = 0.011) as compared to TIMP-1 alone (Figure 6C,E). Moreover, ranking patients based on expression levels into quartiles and comparing survival curves between the highest and lowest quartiles shows that for the two-gene signature, the *p*-value improves by order of magnitude from *p* = 0.039 for TIMP-1 only to *p* = 0.0012 for the two-gene signature (Figure 6F). This indicates that the co-expression of TIMP-1 and IL-6 has a negative impact on the survival of lung adenocarcinoma patients. Patients with a higher co-expression of TIMP-1 and IL-6 have a lower survival probability and vice versa.

## 4. Discussion

Although some novel tumor therapies have been developed for lung cancer, chemotherapy, combined with radiation therapy and surgery, remains the mainstay of treatment in NSCLCs [26]. Developing predictive biomarkers and combining novel “molecularly targeted” agents with standard chemotherapy could be promising strategies, particularly to overcome chemotherapy resistance in lung cancer. Circulating TIMP-1 in serum or urine has been regarded as a novel biomarker for the diagnosis and prognosis of various diseases, including cancer [27,28]. Moreover, meta-analysis of clinical data from publicly available databases (TCGA database) of LUAD in this study supports TIMP-1 being an important potential biomarker in lung cancer, as significantly elevated levels of TIMP-1 are seen in NSCLC tissues in comparison to benign lung tissue. This also raises the possibility that TIMP-1 could also be a possible diagnostic marker. 

Several studies have documented the significant association of high TIMP-1 serum levels with poor response to chemotherapy [29,30,31]. The results of our apoptosis studies provides direct evidence to prove the critical role of TIMP-1 in resisting the response to chemotherapy. Genetically silencing TIMP-1 remarkably increased sensitivity to frontline chemotherapeutic agents in NSCLC cells. However, there has been no clear mechanistic approach defining how TIMP-1 causes chemoresistance. Therefore, we explored molecular mechanisms by which TIMP-1 initiates chemoresistance in NSCLCs. As an initial step, we excluded the influence of ATP-binding cassette (ABC) transporters from TIMP-1-mediated chemoresistance due to the inverse overexpression or no change of the MDR1 gene in TIMP-1 KD clones. This led us to identify IL-6 as a potential mediator involved in resistance, based on multiplex screening of secreted factors. IL-6 is a well-documented pleiotropic cytokine with an established role in cancer progression and therapeutic resistance [13]. Our follow-up apoptosis rescue assay indicated that exogenous rhIL-6 or rhTIMP-1 rescued KD clones from chemo-induced apoptosis, defining TIMP-1 and IL-6 being key determinants contributing to chemoresistance in NSCLCs. Furthermore, increased expression of TIMP-1 and IL-6 in chemoresistant-selected clones reinforces their importance in this process. In a complex system such as this, it is virtually impossible to completely rule out possible interactions of other factors within the ECM including MMP-dependent factors like hyaluronan and tenascin, might participate in the procedure of TIMP-1 regulation of IL-6 [12,32,33]. The current study; however, sought to primarily examine the TIMP-1/IL-6 relationship. 

STAT3 is a pivotal downstream effector of IL-6, activated via Janus kinase (JAK) upon IL-6 binding to its cognate receptors [34]. Sustained activation of STAT3 signaling, along with IL-6 production in culture medium, indicates that IL-6 might play a role in resistance in NSCLC. However, it should be noted that in our studies, exogenous rhTIMP-1 or rhIL6 could not completely rescue NSCLC cells from chemo-induced apoptosis. This is consistent with the fact that TIMP-1 knockdown, to a certain extent, increased drug sensitivity in NSCLCs. Thus, it appears that soluble factors are critical to create conditions for tumor progression subsequent to chemotherapy.

Our studies provide strong evidence for the first time that the regulation of IL-6 expression is modulated by TIMP-1 in NSCLCs. As part of this novel finding, we have also shown a bona fide positive correlation between TIMP-1 and IL-6 under varied culture conditions. A previous study has shown that IL-6 and TIMP-1 contribute to the chemoresistant niche promoting survival of tumor cells in a murine model of Burkitt’s lymphoma; however, the direct relationship between TIMP-1 and IL-6 remained unexplored [16]. In the present study, IL-6 was significantly shown to be modulated by TIMP-1 since genetically silencing TIMP-1 significantly decreased IL-6 mRNA and protein levels under normoxia, as well as hypoxia, the latter being a known critical player in tumor progression [35]. Similarly, specific blockage of secreted TIMP-1 with neutralizing anti-TIMP-1 antibodies delayed the elevation of IL-6 mRNA and protein over the time course of cultures, suggesting IL-6 yield, at least partially, depends on secreted TIMP-1 product. To unequivocally prove TIMP-1 regulation of IL-6, we have shown that exogenous TIMP-1 markedly induced IL-6 transcription in NSCLC cells in a dose-dependent manner. Moreover, during this induction, TIMP-1 enhanced the transcriptional activity of AP-1, which resulted in upregulated IL-6 production. However, knockdown of IL-6 cannot significantly alter TIMP-1 expression in H460 cells, which is inconsistent with prior conclusions of TIMP-1 being downstream of IL-6 [36,37,38]. This suggests different regulatory mechanisms may be applicable between TIMP-1 and IL-6 expression depending upon elements present within various cell types or cancer specific tumor microenvironments, and our results may be specific to this interaction within NSCLC.

NFκB and AP-1 are known to be two primary transcription factors responsible for IL-6 regulation [24]. In our analysis of TIMP-1 KD versus NT clones, we found AP-1 was positively associated with IL-6 expression, but NFκB was inversely correlated. This paradoxical observation of NFκB P65 may be a compensatory feedback mechanism to overcome loss of IL-6 along with TIMP-1 knockdown since the NF-kB-IL-6-STAT3 cascade is an important regulator of tumor cell proliferation and survival [34].

The changes in AP-1 (c-Jun) signaling in NSCLCs after short-interval treatments with exogenous TIMP-1, combined with knockdown of TIMP-1, demonstrated that AP-1 might be the real transcription factor responsible for the TIMP-1 regulation of IL-6. Alternatively, additional evidence from protein-DNA interaction techniques, like chromatin immunoprecipitation sequencing (ChIP-seq) or ChIP-qPCR, may be required to prove their direct binding. Interestingly, AP-1 is also known to be one of the important players in TIMP-1 transcription [39]. However, increased transcriptional activities as seen in the phosphorylation of AP-1(c-Jun) and NFκB (P65) subunits in chemo-resistant cells suggests a different regulatory mechanism to respond to chemotherapy in NSCLCs from that in non-stress conditions. Although chemotherapeutic treatment still induced IL-6 upregulation in TIMP-1 KD clones, knockdown of TIMP-1 significantly decreased the basal level of IL-6 compared to NT clones. Therefore, TIMP-1 remains a critical element to determine the constitutional level of IL-6 in NSCLC. 

Our interrogation of the TCGA database does indeed confirm a close relationship between TIMP-1 expression and co-expression of IL-6 as presented above. This relationship of IL-6 with TIMP-1 expression was evident in primary tumor tissues of lung adenocarcinoma. However, the IL-6 mRNA level is significantly higher in adjacent normal tissue than in tumor tissue, and single gene analysis of IL-6 in survival probability of LUAD patients is not significant (data not shown). This paradoxical finding may be attributed to the complex contextual nature of the tumor whereby many producers of IL-6 are various elements within the TME, particularly stromal and immune cells, such as macrophages, monocytes and inflammatory T cells [40]. However, if combined with the expression of TIMP-1 in tumors, co-expression of the two-gene signature (TIMP-1 and IL-6) became related to the probability of surviving in comparison to the IL-6 single gene, and even more significant than the single gene analysis of TIMP-1 within LUAD patients. 

## 5. Conclusions

High serum and tissue expression of TIMP-1 is associated with poor prognosis and decreased survival for several cancers including lung cancer. The current study defines an important and novel role for TIMP-1 in chemoresistance in NSCLC via regulation of IL-6 expression. Using two frontline chemotherapy drugs against NSCLC, we have identified the mechanism involved in TIMP-1’s chemoresistance function. This study for the first time provides evidence that TIMP-1 modulates IL-6 and activates downstream signaling. Interrogation of the TCGA database allowed us to confirm this close relationship between TIMP-1 and Il-6 co-expression with LUAD patient survival and the significance of this two-gene signature. Targeting the TIMP-1/IL6 axis in NSCLCs may; therefore, provide an innovative approach to enhance the efficacy of chemotherapy, thus leading to better clinical outcomes in this devastating disease.

## Supplementary Materials

The following are available online at https://www.mdpi.com/2072-6694/11/8/1184/s1, Figure S1: Effects of TIMP-1 gene knockdown on Gemcitabine-induced apoptosis in NSCLC cells. Figure S2: Effects of TIMP-1 gene knockdown on Cisplatin-induced apoptosis in NSCLC cells. Figure S3: Effects of TIMP-1 gene knockdown on the gene transcription of inflammatory cytokines in NSCLC cells. Figure S4: Expression profiles of TIMP-1 in NSCLC cell lines. Figure S5: Cell proliferation and viability measurement by MTT assay. Figure S6: Original Western blot images. Table S1: Primer Sequences, Table S2: List of Antibodies.
