# Supplementary material for: TIMP-1-Mediated Chemoresistance via Induction of IL-6 in NSCLC
Source: Cancers (Basel). 2019 Aug 15;11(8):1184. doi: 10.3390/cancers11081184 (PMC6721590; doi:10.3390/cancers11081184)
Supplement: Supplementary file 1 [file cancers-11-01184-s001.pdf]

cancers

Supplementary Materials: TIMP-1-Mediated Chemoresistance via Induction of IL-6 in NSCLC

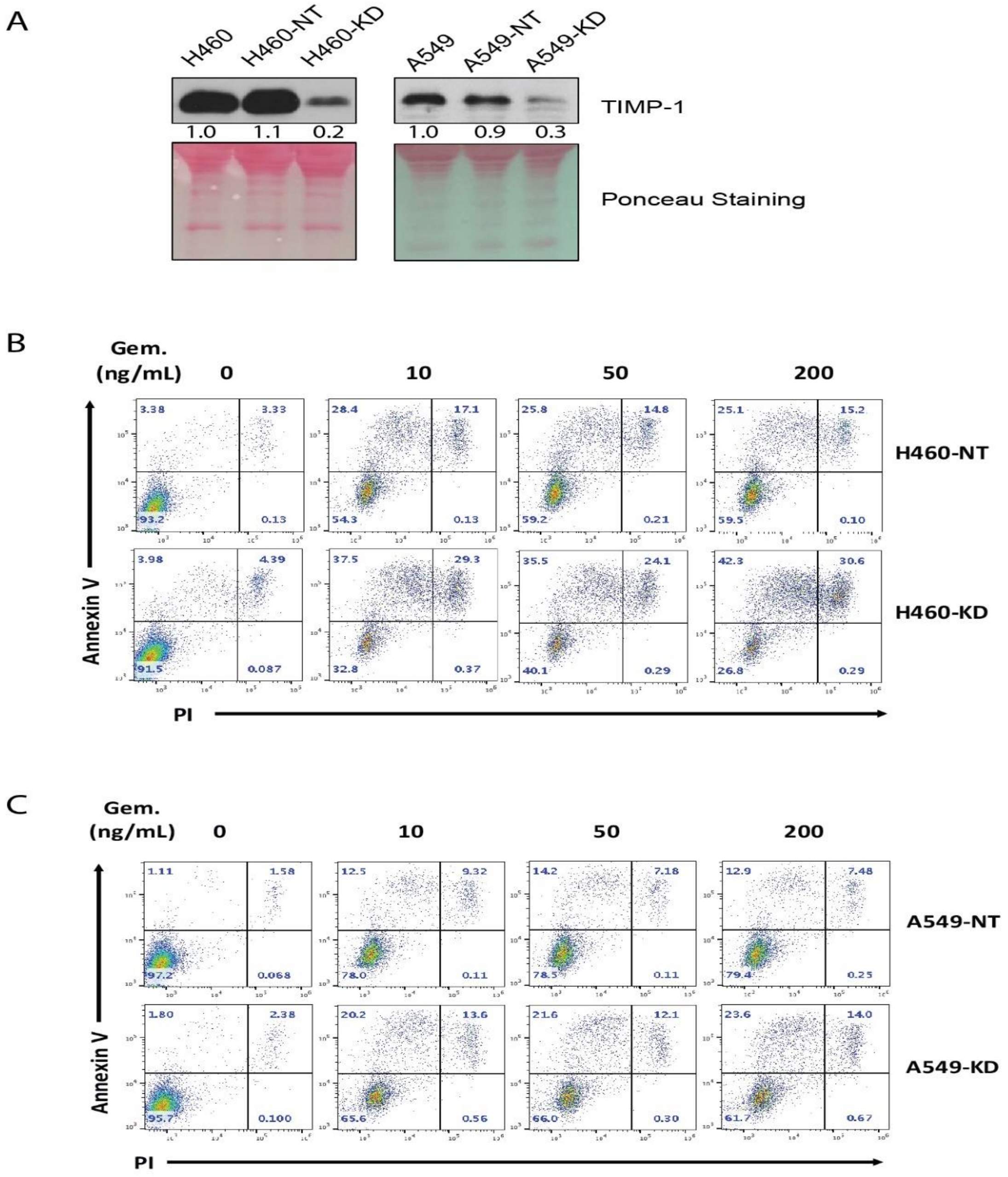

Figure S1. Effects of TIMP-1 gene knockdown on Gemcitabine-induced apoptosis in NSCLC cells. Same counts of NSCLC cells (A549 and H460) and derived TIMP-1 KD and NT clones were seeded and cultured in complete medium for two days. Equal volumes of supernatants were loaded for human TIMP-1-specific immunoblotting analysis. (**B) and** (**C**) Human NSCLC cells (A549 and H460) encoding non-target scrambled shRNA (NT) sequence or TIMP-1-specific knockdown shRNA (KD) sequence were seeded in 24-well plates (3 × 10^4/well). At log-phase, cells were treated with variable doses of gemcitabine as indicated. All floating and adherent cells were collected at 72 hours post treatment, stained with Annexin V and PI and analyzed by flow cytometry. Representative data are shown for apoptosis and cell death of H460 (**B**) and A549-derived cells (**C**).

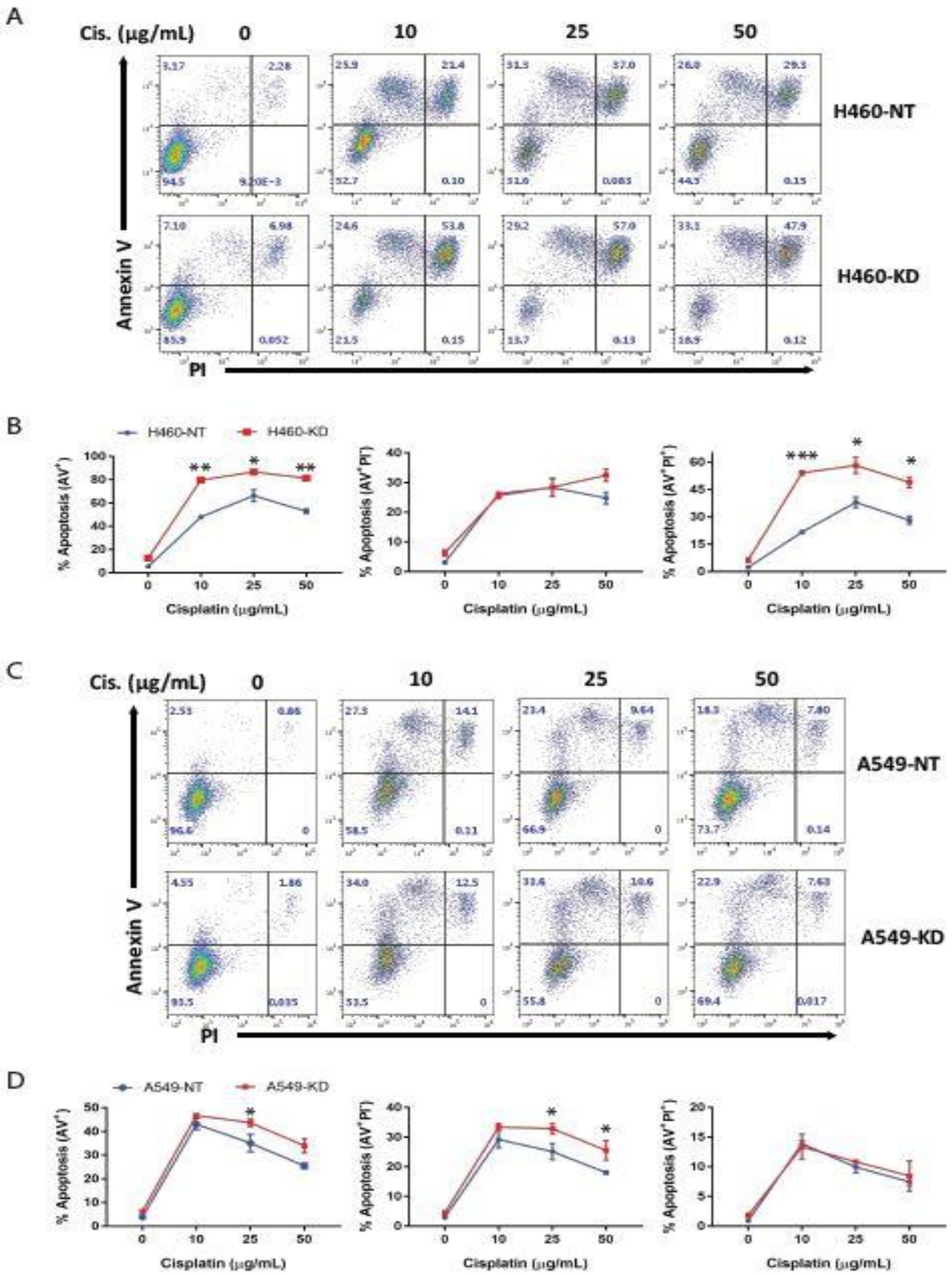

Figure S2. Effects of TIMP-1 gene knockdown on Cisplatin-induced apoptosis in NSCLC cells. Human NSCLC cells (A549 and H460) encoding non-target scrambled shRNA (NT) sequence or TIMP-1-specific knockdown shRNA (KD) sequence were seeded on 24-well plates (3×10^4/well). At log-phase, cells were treated with variable doses of Cisplatin as indicated. All floating and adherent cells were collected at 48 hours post treatment, and analyzed by flow cytometry following Annexin V and PI staining. Representative data are shown for apoptosis and cell death of H460 and A549-derived cells (**A, C**). Statistical analysis of total apoptosis (Annexin V^+), early apoptosis (Annexin V^+PI^-) and apoptotic cell death (Annexin V^+PI^+) is shown for H460-derived cells (**B**) and A549-derived cells (**D**). Data shown is representative from one of two independent experiments.

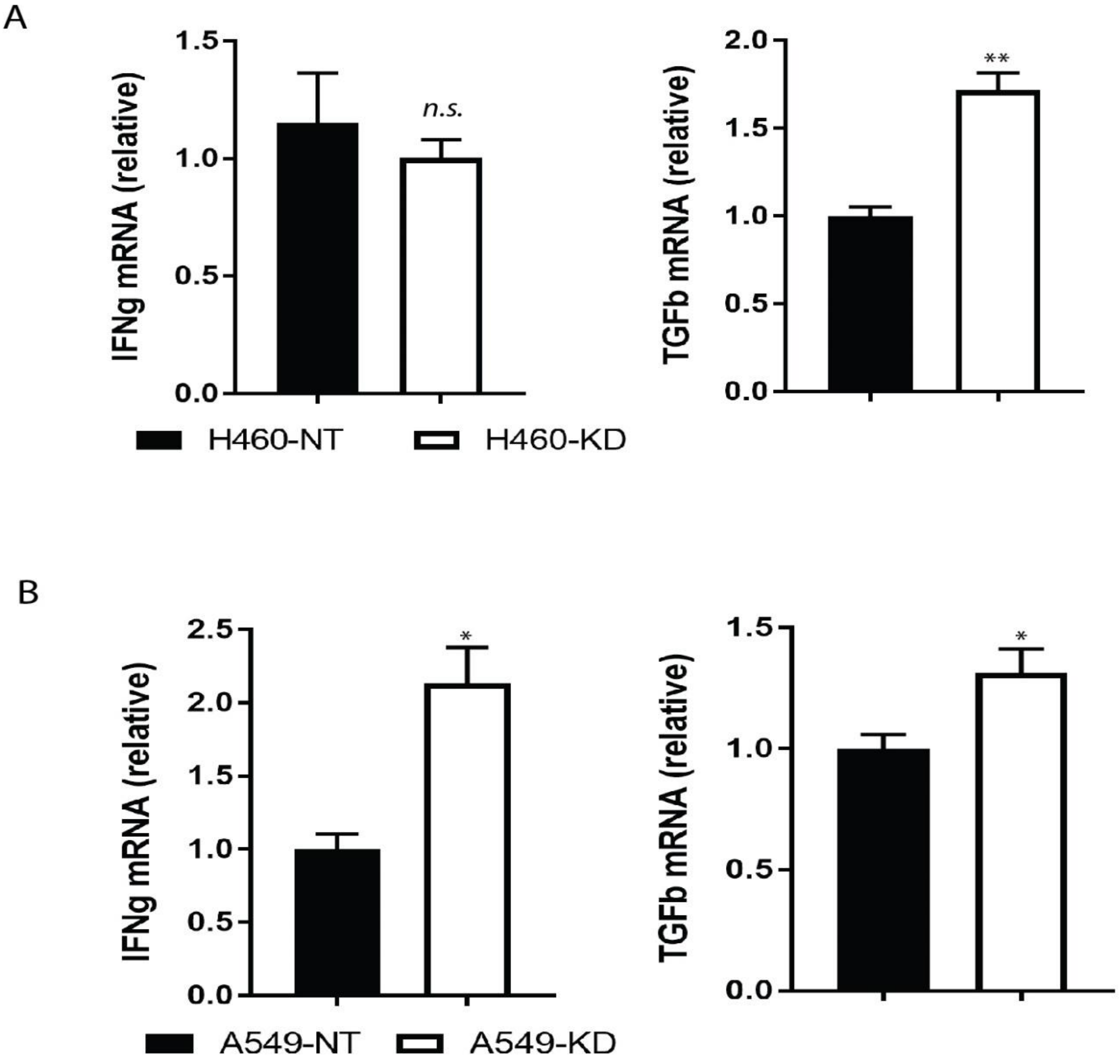

Figure S3. Effects of TIMP-1 gene knockdown on the gene transcription of inflammatory cytokines in NSCLC cells. mRNA levels of IFNγ and TGFβ were determined in H460- (**A**) or A549- (**B**) derived shRNA-encoding cells (A549-NT, A549-KD, H460-NT and H460-KD) by real-time qRT-PCR.

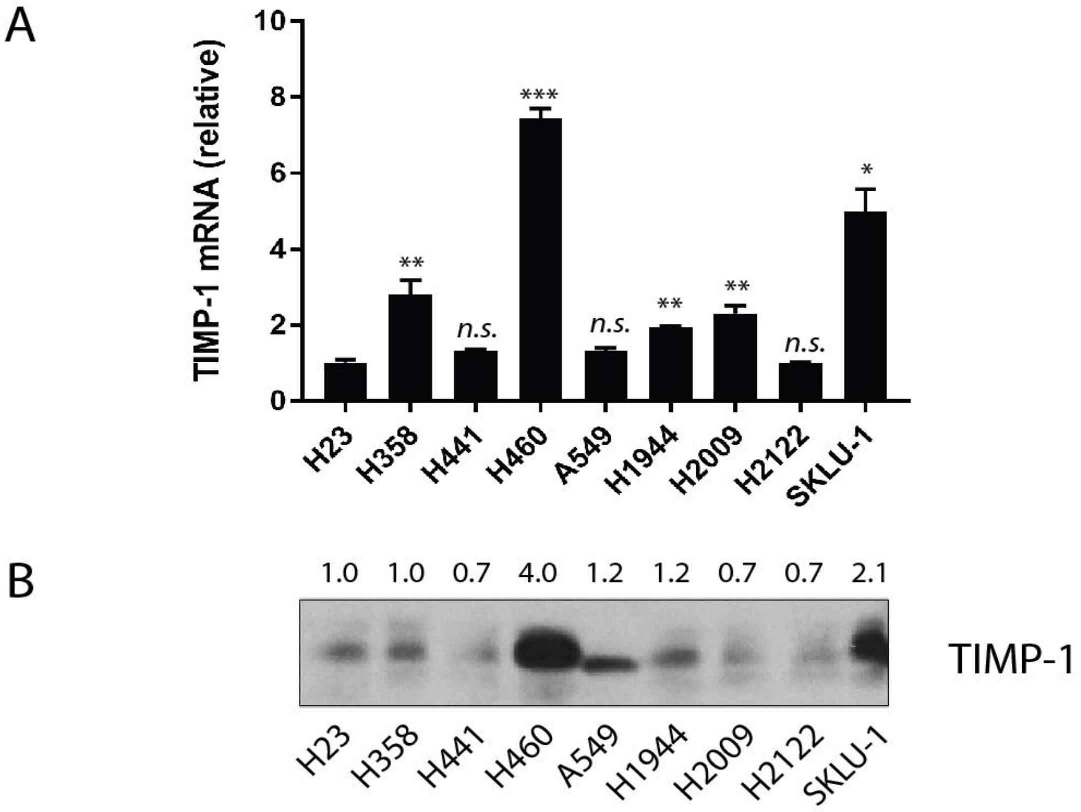

Figure S4. Expression profiles of TIMP-1 in NSCLC cell lines. (**A**) mRNA levels of TIMP-1 were determined in various NSCLC cell lines, including H23, H358, H441, H460, A549, H1944, H2009, H2122, SKLU-1, by real-time qRT-PCR. (**B**) Supernatants of culture media from above cell cultures were collected and checked for TIMP-1 protein levels by immunoblotting analysis.

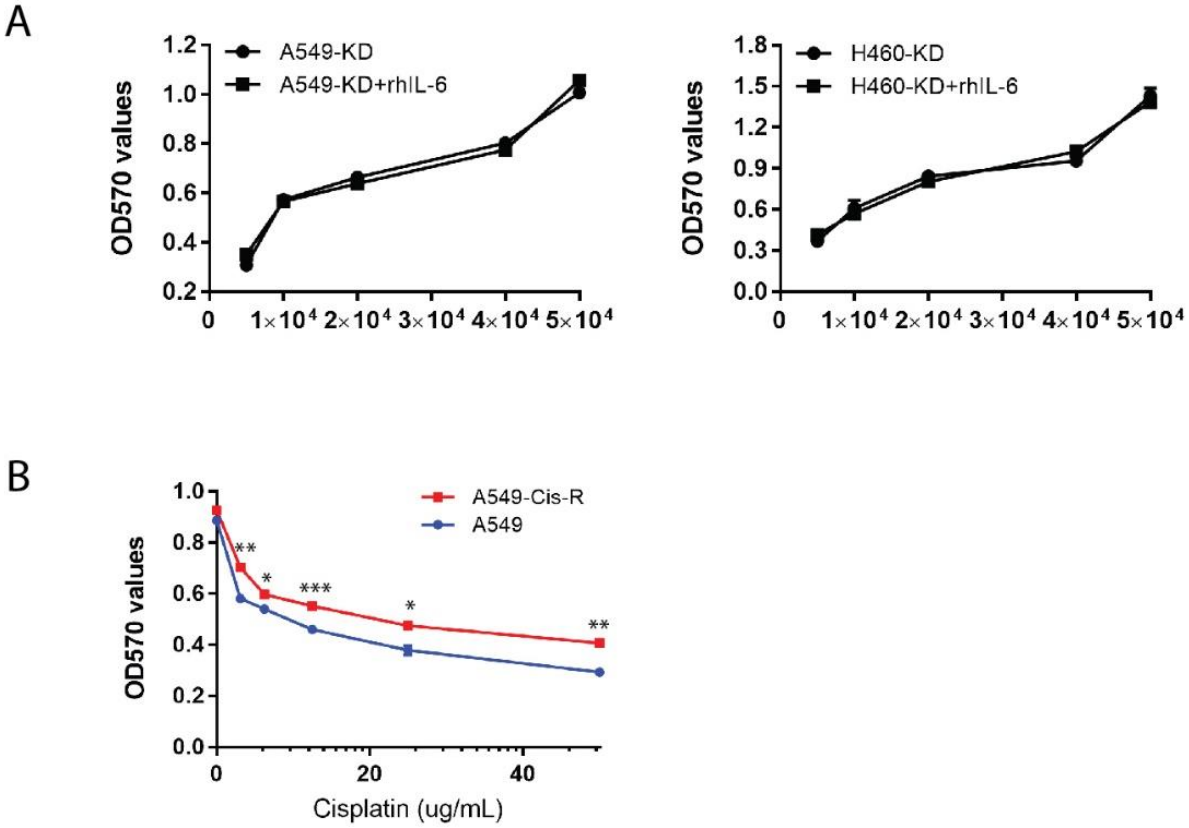

Figure S5. Cell proliferation and viability measurement by MTT assay. (**A**) Exponentially growing A549-KD and H460-KD cells were seeded in 96-well plates at different densities (cells/mL) as indicated in complete culture medium with or without rhIL-6 (25 ng/mL). Forty-eight hours later, cells were measured with MTT reagent according to the instruction of the kit (Vybrant® MTT Cell Proliferation Assay Kit, Invitrogen). (**B**) Equal numbers of A549 and A549-Cis-R cells (1×10^4/well) were seeded in 96-well plates in 100 μL complete culture medium with different doses of Cisplatin as indicated. Forty-eight hours later, cells were assessed by MTT assays. * $p < 0.05$; ** $p < 0.01$; *** $p < 0.001$.

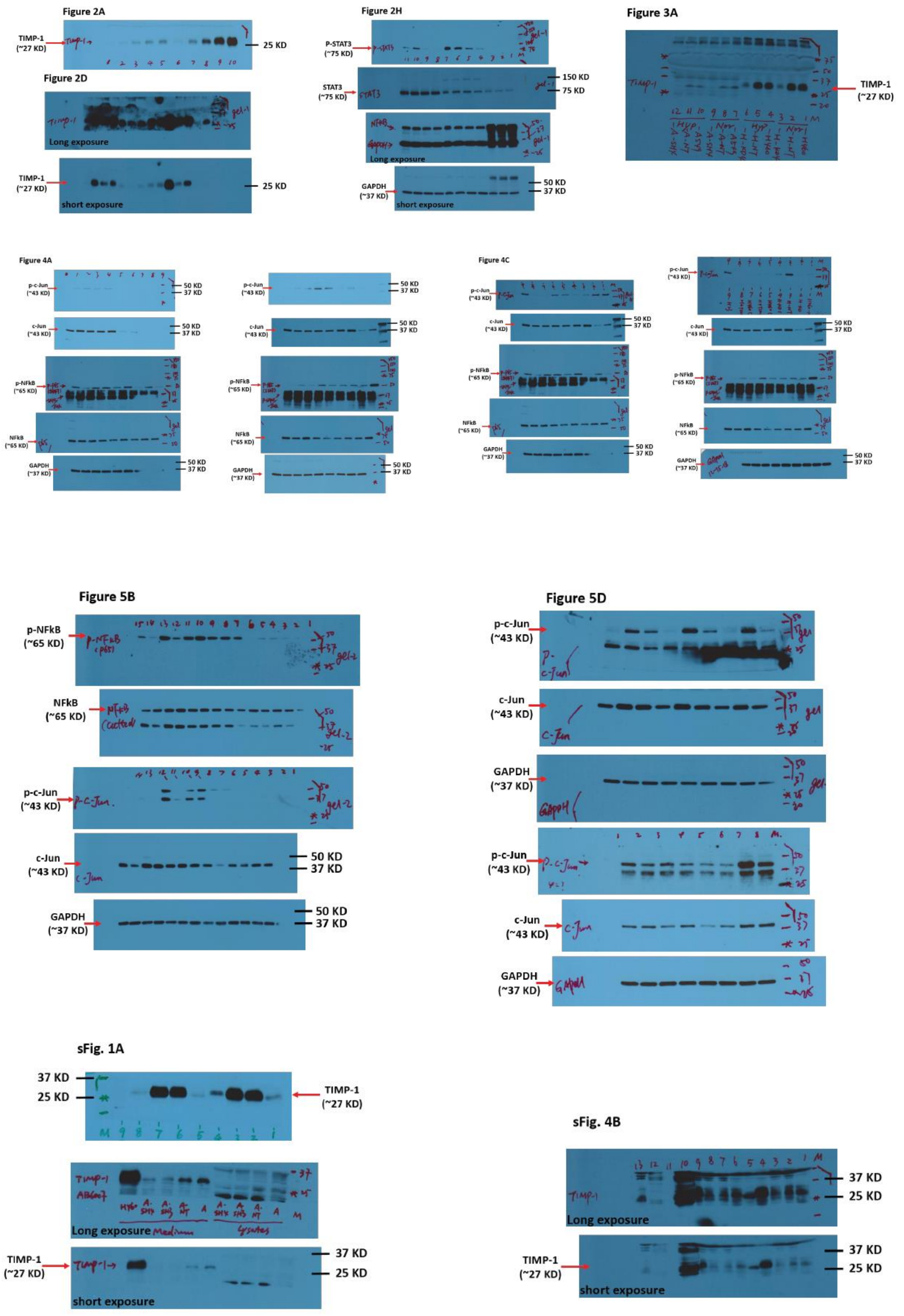

Figure S6. Original Western blot images.

Table S1. Primer Sequences.

	Primers	Sequences (5′-3′)
1	IL-10_Forward	GCTGGAGGACTTTAAGGGTTAC
2	IL-10_Reverse	GATGTCTGGGTCTTGGTTCTC
3	IL-6_Forward	CCAGGAGAAGATTCCAAAGATGTA
4	IL-6_Reverse	CGTCGAGGATGTACCGAATTT
5	TGF-β_Forward	CGTGGAGCTGTACCAGAAATAC
6	TGF-β_Reverse	CACAACTCCGGTGACATCAA
7	IFNγ_Forward	ATGTCCAACGCAAAGCAATAC
8	IFNγ_Reverse	ACCTCGAAACAGCATCTGAC
9	TNF-α_Forward	CCAGGGACCTCTCTCTAATCA
10	TNF-α_Reverse	TCAGCTTGAGGGTTTGCTAC
11	TIMP-1_Forward	ATGGACTCTTGCACATCACTAC
12	TIMP-1_Reverse	GGGATGGATAAACAGGGAAACA
13	ABCB1_Forward	TGCTGGTTGCTGCTTACA
14	ABCB1_Reverse	GCCTATCTCCTGTCGCATTATAG
15	β-Actin_Forward	CACTCTTCCAGCCTTCCTTC
16	β-Actin_Reverse	GTACAGGTCTTTGCGGATGT

Table S2. List of Antibodies.

	Targeted Antigens	Antibody Types (clone#/Cat#)	Companies	Dilutions
1	TIMP-1	pAB (#AB770)	Millipore Sigma	1:1000
2	phosphor-STAT3 (Tyr705)	mAb (D3A7)	Cell Signaling Technology	1:1000
3	STAT3	mAb (124H6)	Cell Signaling Technology	1:1000
4	phosphor-c-Jun (Ser63)	pAB (#9261)	Cell Signaling Technology	1:1000
5	c-Jun	mAb (60A8)	Cell Signaling Technology	1:1000
6	phosphor-NFκB P65 (Ser547)	pAb (#ABS403)	Millipore Sigma	1:2000
7	NFκB P65	mAb (D14E12)	Cell Signaling Technology	1:1000
8	GAPDH	mAB (6C5)	Santa Cruz Biotechnology	1:500
